# UCH-L1 and UCH-L3 regulate the cancer stem cell-like properties through PI3 K/Akt signaling pathway in prostate cancer cells

**DOI:** 10.1080/19768354.2021.1987320

**Published:** 2021-10-11

**Authors:** Jae Eun Lee, Yun Hwan Lim, Jung Hwa Kim

**Affiliations:** Department of Biological Sciences, Inha University, Incheon, Korea

**Keywords:** UCH-L1, UCH-L3, CSC-like property, PI3 K/akt signaling pathway

## Abstract

Castration-resistant prostate cancer (CRPC) is a highly aggressive and advanced prostate cancer that is currently incurable with conventional therapies. The recurrence and chemotherapy-resistant properties of CRPC are attributed to prostate cancer stem cells (CSCs). On the other hand, the factors regulating the prostate CSC-like properties have not been studied extensively. Previously, ubiquitin C-terminal hydrolase-L1 (UCH-L1) and ubiquitin C-terminal hydrolase-L3 (UCH-L3) were reported to be involved in prostate cancer cell progression through the epithelial-to-mesenchymal transition (EMT) regulation. Here, the differential regulation on the CSC-like properties by UCH-L1 and UCH-L3 were identified in prostate cancer cells. The CSC-like characteristics, such as the expression of pluripotency markers, chemoresistance, and sphere-forming ability, were promoted by UCH-L1, whereas those were repressed by UCH-L3. Moreover, the modulation of CSC-like properties by UCH-L1 and UCH-L3 was through the PI3 K/Akt signaling pathway. The CSC-like properties induced by UCH-L1 overexpression or UCH-L3 depletion were suppressed by the PI3 K/Akt pathway inhibitor. In conclusion, UCH-L1 and UCH-L3 are novel regulators of the CSC-like properties and shed light on new therapeutic strategies to overcome CSCs in prostate cancers.

## Introduction

Prostate cancer is the most prevalent and major life-threatening cancer among men in western countries (Siegel et al. 2015). In Korea, the incidence of prostate cancer has increased similarly to western countries due to lifestyle changes (Kimura and Egawa 2018). Castration-resistant prostate cancer (CRPC), a highly aggressive and advanced prostate cancer, is currently incurable with conventional therapies and is a major clinical challenge. Prostate cancer consists of a highly heterogeneous population of cells. In the heterogeneous pool of tumor cells, cancer stem cells (CSCs) are very few in number and are characterized by their ability to self-renew and initiate tumors. Many studies reported that the relapse and chemotherapy-resistant properties of CRPC are attributed to CSCs (Ojo et al. 2015).

Ubiquitin C-terminal hydrolase-L1 (UCH-L1) and ubiquitin C-terminal hydrolase-L3 (UCH-L3) are deubiquitinating enzymes (DUBs) that remove ubiquitin (Ub) from the target proteins (Kim et al. 2003). The amino acid identity and structure in UCH-L1 and UCH-L3 are similar, but the biochemical activities are somewhat different except for the Ub hydrolyzing activity. Interestingly, UCH-L1 obtains Ub ligating activity by forming a dimer. One particular feature of UCH-L3 is its hydrolase specificity for both Ub and Nedd8. UCH-L1 and UCH-L3 are involved in the progression of many tumors, including lung, breast, prostate, and pancreatic cancers (Tezel et al. 2000; Miyoshi et al. 2006; Jang et al. 2011; Song et al. 2014).

During tumor progression, mobility-free epithelial cells are transformed into mobile mesenchymal cells through an epithelial-to-mesenchymal transition (EMT). Recent studies have proposed that EMT induces CSC-like features in cancer cells. During cancer development, activation of the EMT program increases the tumor initiation capacity and imparts cancer cells with resistance to a range of therapeutic agents (Shibue and Weinberg 2017; Zhou et al. 2020). Previously, the EMT regulation of prostate cancer cells by UCH-L1 and UCH-L3 was reported (Jang et al. 2011; Song et al. 2014). UCH-L1 induced EMT to increase the invasion and metastasis of prostate cancer cells. On the other hand, UCH-L3 inhibited EMT and had the opposite effect of UCH-L1.

The view that UCH-L1 and UCH-L3 regulate EMT suggests that these two DUBs might also be associated with the regulatory mechanism of the CSC-like characteristics in prostate cancer cells. This study examined the differential regulation of the CSC-like properties by UCH-L1 and UCH-L3 in prostate cancer cells. UCH-L1 increased the CSC-like properties, whereas UCH-L3 had an opposite action in prostate cancer cells. In addition, UCH-L1 and UCH-L3 regulate the CSC-like properties by modulating the PI3K/Akt signaling pathway. These findings suggest that UCH-L1 and UCH-L3 are novel regulators that modulate the CSC-like properties and might provide new therapeutic strategies to treat advanced prostate cancers.

## Material and methods

### Cell lines and culture media

DU145 cells were grown in RPMI 1640 (Welgene). RWPE1 cells were maintained in Keratinocyte-serum-free medium (K-SFM, Invitrogen) containing EGF (1.25 g/L) and bovine pituitary extract (BPE) (12.5 mg/L). RPMI1640 and K-SFM were supplemented with 10% FBS and 0.1 mg/mL streptomycin plus 100 U/mL penicillin.

### Antibodies and chemicals

The antibodies used and their manufacturers are as follows. OCT4 (sc-9081), BMI1 (sc-390443), SOX2 (sc-17320), Nanog (sc-134218), AKT (sc-8312), 4EBP1 (sc-9977), p70 S6 kinase (sc-8418), and UCH-L1 (sc-58593) were purchased from Santa Cruz. KLF4 (ab151733), phospho-AKT (ab81283), and UCH-L3 (ab87154) were obtained from Abcam. Phospho-4EBP1 (2855) and phospho-p70 S6 kinase (9205) were purchased from Cell signaling. Flag M2 (F3165) and β-actin (A1978) were supplied by Sigma-Aldrich. The following chemicals were used: BEZ235 (MedChem Express, HY-50673), LY294002 (Cayman, 70920), doxorubicin (Sigma, D1515), etoposide (Sigma, E1383), and mitoxantrone dihydrochloride (MTX) (Sigma, M6545).

### Generation of stable overexpression and knockdown cell lines

The procedures for the stable overexpression of UCH-L1, UCH-L1 C90S, UCH-L3, or UCH-L3 C95S and the knockdown of UCH-L1 or UCH-L3 in RWPE1 or DU145 were referenced from previous reports (Jang et al. 2011; Song et al. 2014). The pMSCVpuro empty vector was used as a control for the experiments using stable cell lines.

### Real-time RT–PCR and primers

After extracting the total RNA using a TRIzol reagent, the first cDNA strand was synthesized with the oligo (dT) primers and RevertAid reverse transcriptase. The SYBR green and QuantStudio 1 Real-Time PCR System was used for the semiquantitative real-time RT–PCR experiments. The data were analyzed using the ΔΔCt method and was normalized to the GAPDH gene. The sequences of the primers used in the study were referenced as follows: *Oct4, Klf4, Sox2* (Yin et al. 2013), *Bmi1* (Wellner et al. 2009), *Nanog* (Freberg et al. 2007), *UCH-L1* (Jang et al. 2011), *UCH-L3* (Song et al. 2014), *ABCG2* (Tomiyasu et al. 2013), *CD44* (Swarts et al. 2013), and *CD133* (Le et al. 2013). All reactions were performed in triplicate.

### Sphere forming assay

After isolating the RWPE1 and DU145 cells into single cells, 100 cells were seeded in 96-well Ultra-low Attachment plates. The media for the sphere forming assay was serum-free DMEM/F12 K medium containing B27, 20 ng/mL EGF and bFGF, and 4 μg/mL insulin. After 14 days for RWPE1 or 7 days for DU145, the number of spheres with a diameter exceeding 100 μm was counted under a microscope at 200× magnification.

### The Cancer genome atlas (TCGA) data analysis

The TCGA-PRAD clinical data and RNA sequencing data were downloaded from the cBioPortal. The samples from 491 patients were examined with the mRNA levels of UCH-L1 and UCH-L3 to classify them into low- (n = 245) and high-expression groups (n = 246) using the median expression level as the cut-off for each mRNA. For statistical analysis, the log-rank test was used. The Kaplan-Meier method was used to obtain the disease-free survival curves using R software.

## Results

### UCH-L1 and UCH-L3 regulate the expression of the CSC pluripotency markers

**Figure 1:**
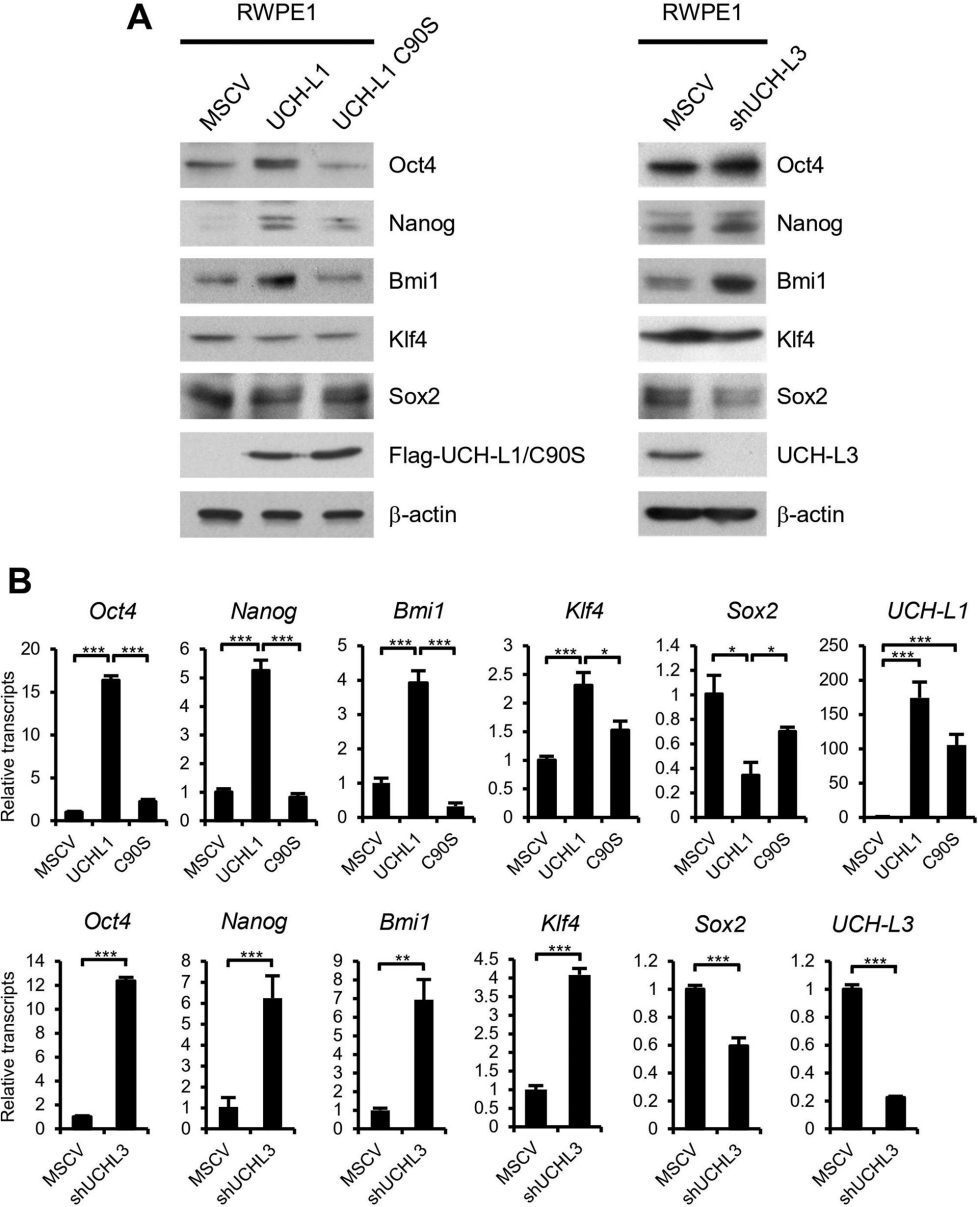
. UCH-L1 and UCH-L3 regulate the pluripotency markers in RWPE1 cells. (A) The expression of pluripotency markers, including Oct4, Nanog, Bmi1, Klf4, and Sox2, were examined by immunoblotting in RWPE1 stable cell lines as indicated. β-actin was used as the loading control. (B) Real-time quantitative RT-PCR analysis of *Oct4, Nanog, Bmi1, Klf4,* and *Sox2* in RWPE1 stable cell lines. The values shown are the mean ± SD of three independent experiments, and the *p*-value was obtained from a Student’s t-test. *p < 0.05, **p < 0.01, ***p < 0.001.

As EMT progresses, cancer cells undergo dedifferentiation and adopt a CSC status (Shibue and Weinberg 2017). The involvement of UCH-L1 and UCH-L3 in the invasion and metastasis of prostate cancer cells via EMT regulation has been reported. The concept that UCH-L1 and UCH-L3 regulate EMT suggests that they might help modulate the CSC-like properties in prostate cancer cells. In our previous reports, the UCH-L1 and UCH-L3 expression pattern was opposite in RWPE1 benign prostate tumor cells. In RWPE1, UCH-L1 was barely expressed but UCH-L3 expression was high (Jang et al. 2011; Song et al. 2014). RWPE1 cells, in which the expression of each enzyme was reversed by genetic manipulation, were used to examine the role of UCH-L1 and UCH-L3 on the modulation of the CSC-like characteristics. That is, a cell line that overexpressed the UCH-L1 or UCH-L1 active site mutant and a cell that knocked down UCH-L3 were generated. First, the expression of the pluripotent stem cell markers was checked in these cells. Interestingly, UCH-L1 overexpression and UCH-L3 knockdown induced several pluripotent stem cell markers, such as Oct4, Nanog, and BMI1, at the protein and mRNA levels (Figure 1). Among the pluripotency markers tested, Klf4 and Sox2 showed an inconsistent pattern between the protein and mRNA level. UCH-L1 C90S, which is devoid of the Ub hydrolase activity by substituting cysteine 90 to serine, failed to induce the pluripotency markers. These results suggest that the pluripotency markers are regulated partially by UCH-L1 and UCH-L3 in RWPE1 cells.

### UCH-L1 and UCH-L3 regulate the CSC-like behaviors in RWPE1 cells

**Figure 2.**
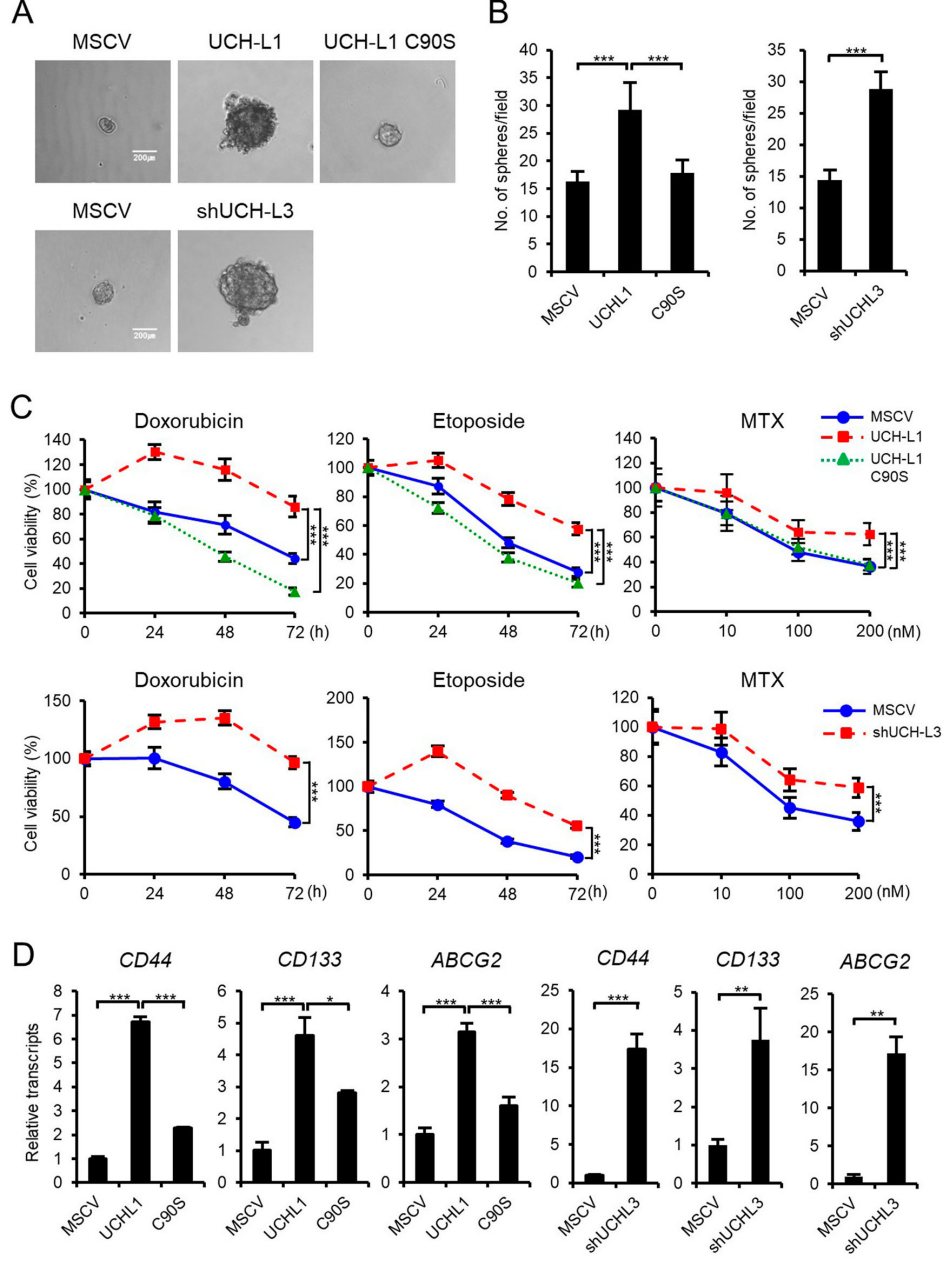
UCH-L1 and UCH-L3 regulate the CSC-like behaviors in RWPE1 cells. (A) The self-renewal capacity of each cell was evaluated using a sphere forming assay. An equal number of each stable cell was cultured in 96-well ultra-low attachment dishes for 14 days. The figure shows a representative image from each cell, and the scale bar corresponds to 200 μm was adjusted to all images. (B) The number of spheres with a diameter greater than 100 μm per field was counted. (C) The cell viability of each stable cell was monitored in a time-dependent manner after treating 0.5 μg/mL doxorubicin and 50 μM etoposide or in a dose-dependent manner after treating MTX for 48 h. (D) Real-time quantitative RT-PCR analysis of *CD44, CD133*, and *ABCG2* in RWPE1 stable cells. The values shown are the mean ± SD of three independent experiments. The *p*-value was obtained using a Student’s t-test. *p < 0.05, **p < 0.01, ***p < 0.001.

The implications of UCH-L1 and UCH-L3 on the CSC-like property modulation were verified by examining their effect on the self-renewal capacity, a defining property of CSCs, using sphere formation assays. UCH-L1 overexpression increased the size and number of spheres depending on the Ub cleavage activity compared to the control cells (Figure 2(A and B)). UCH-L3 depletion also increased the size and number of spheres in RWPE1 cells. Prostate cancer can survive exposure to chemotherapeutic agents through CSCs (Ojo et al. 2015). As expected, UCH-L1 overexpression and UCH-L3 knockdown cells were resistant to drugs, such as doxorubicin, etoposide, and MTX, compared to the control cells (Figure 2(C)). In summary, UCH-L1 promotes CSC-like behaviors, and UCH-L3 has opposite effects. Numerous markers have been proposed for CSCs because of the heterogeneity of prostate tumors. The expression of cell surface markers is often checked to characterize CSCs. Among them, the change in CD44 and CD133 expression, which are used widely to separate and characterize prostate cancer stem cells, were examined (Sharpe et al. 2013). CD44 and CD133 were induced in both UCH-L1 overexpressing or UCH-L3 depleted cells (Figure 2(D)). One of the mechanisms of CSC chemoresistance is the overexpression of the ABC drug transporter, ABCG2 (An and Ongkeko 2009). The correlation between the enhanced chemoresistance by UCH-L1 overexpression or UCH-L3 knockdown and ABCG overexpression was verified (Figure 2(D)).

### UCH-L1 and UCH-L3 regulate the CSC-like properties through the PI3K/Akt signaling pathway

**Figure 3.**
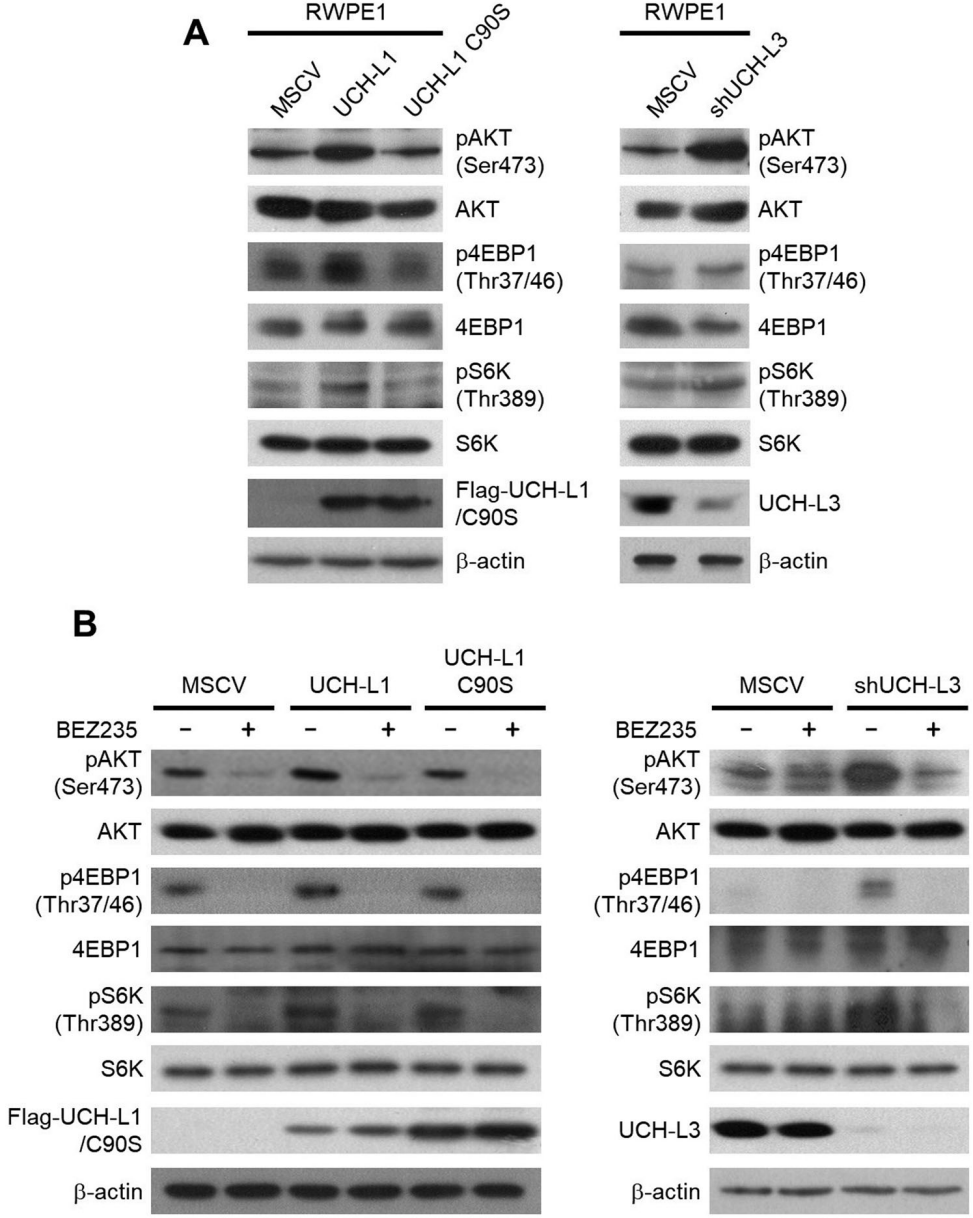
UCH-L1 and UCH-L3 regulate the PI3 K/Akt signaling pathway in RWPE1 cells. (A) The protein levels of several PI3 K/Akt signaling molecules, as indicated in each stable cell, were examined by western blotting. (B) After incubation with DMSO or 200 nM BEZ235 for 48 h, the protein level of several PI3 K/Akt signaling molecules indicated in each stable cell was examined by western blotting.

**Figure 4.**
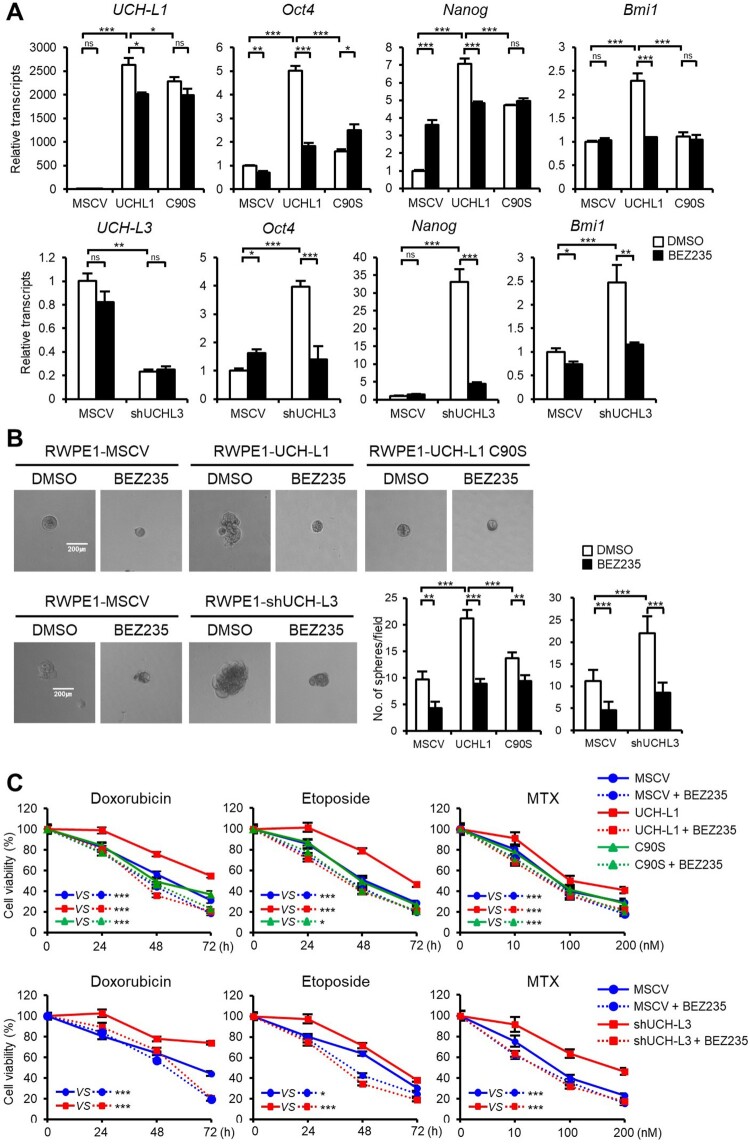
UCH-L1 and UCH-L3 regulate the CSC-like properties through the PI3 K/Akt pathway in RWPE1 cells. (A) Real-time quantitative RT-PCR analysis of *Oct4, Nanog,* and *Bmi1* in DMSO or 200 nM BEZ235 treated RWPE1 stable cell. (B) Sphere forming capacities of each RWPE1 stable cell was measured in 96-well ultra-low attachment dishes after DMSO or 200 nM BEZ235 treatment for 14 days. The figure shows a representative image from each cell, and the scale bar corresponding to 200 μm was adjusted to all images. The number of spheres with a diameter greater than 100 μm per field was counted. (C) The cell viability of each stable cell was measured in a time-dependent manner after treatment with 0.5 μg/mL doxorubicin and 50 μM etoposide or in a dose-dependent manner after treatment with MTX for 48 h in the presence or absence of 200 nM BEZ235. The values shown are the mean ± SD of three independent experiments, and the *p*-value was obtained from a Student’s t-test. *p < 0.05, **p < 0.01, ***p < 0.001.

**Figure 5.**
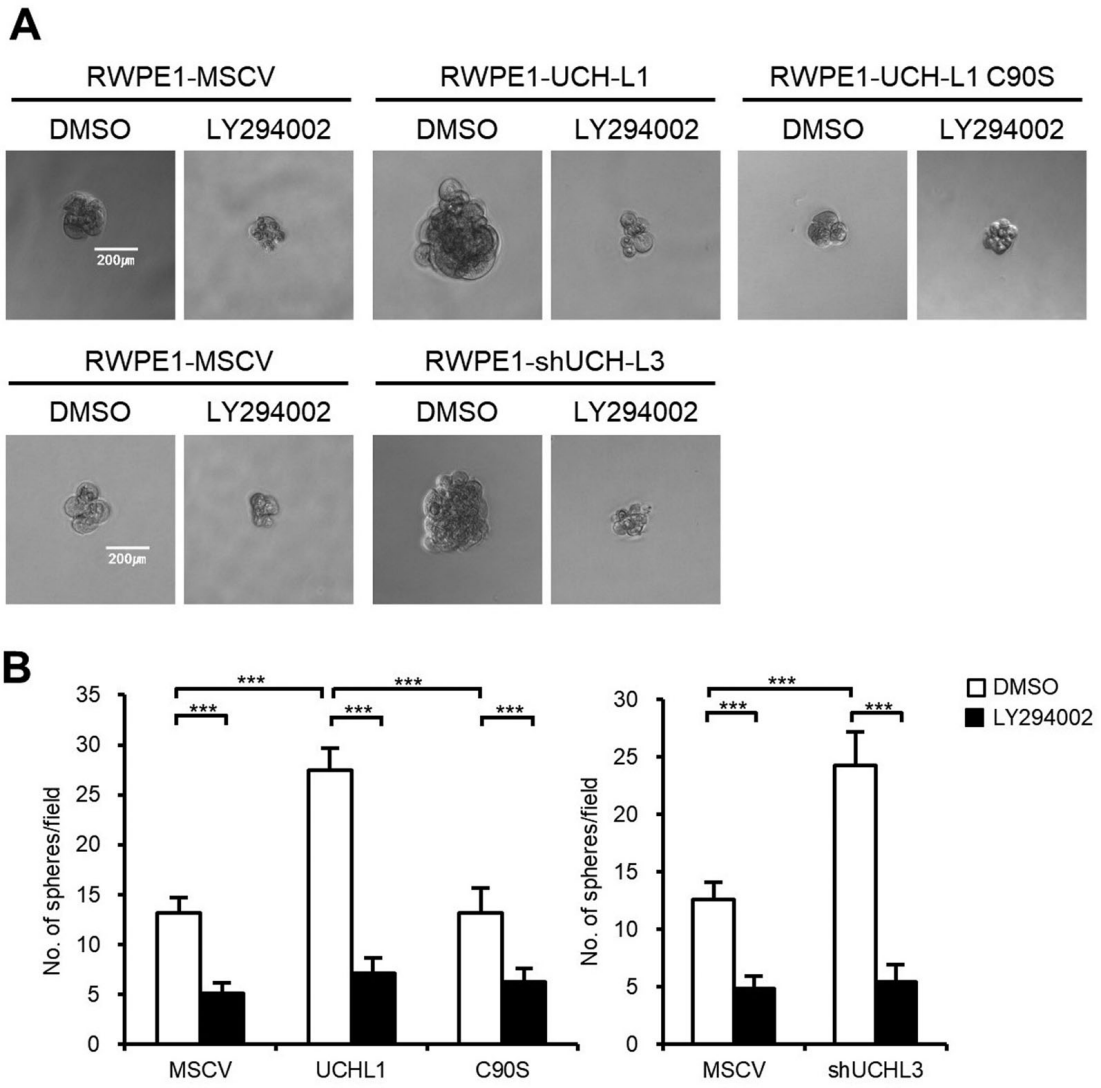
LY294002 inhibits sphere formation in RWPE1 stable cells. (A) Sphere forming capacities of each RWPE1 stable cell was measured in 96-well ultra-low attachment dishes after 50 μg/mL LY294002 treatment for 14 days. The figures show representative images from each cell, and the scale bar corresponding to 200 μm was adjusted to all images. (B) The number of spheres with a diameter greater than 100 μm per field was counted. The values shown are the mean ± SD of three independent experiments, and the *p*-value was obtained using a Student’s t-test. *p < 0.05, **p < 0.01, ***p < 0.001.

Several signaling pathways, including PI3 K/Akt, Wnt/β-catenin, and Notch pathways, are associated with CSCs to help regulate prostate cancer self-renewal, tumor initiation, and chemoresistance (Ni et al. 2014; Cai et al. 2020). Among them, the high prevalence of PI3 K/Akt activation in prostate cancer has made it a promising target to eliminate CSCs and overcome the chemoresistance of castration-resistant prostate cancer. Several downstream targets of the PI3 K signaling pathway were examined in RWPE1 stable cells to determine if UCH-L1- and UCH-L3-induced regulation of CSC phenotypes is mediated through the PI3 K/Akt pathway. Both UCH-L1 overexpression or UCH-L3 knockdown increased the phosphorylation of the downstream targets of the PI3 K/Akt signaling pathway, including pAKT(Ser473), p4EBP1(Thr37/46), and pS6 K(Thr389) (Figure 3(A)). NVP-BEZ235, a dual PI3 K/mTOR inhibitor, was used to confirm the involvement of the PI3 K/Akt signaling pathway in the UCH-L1- and UCH-L3-induced regulation of the CSC-like characteristics. The NVP-BEZ235 treatment inhibited the downstream effectors of the PI3 K/Akt pathway activated by UCH-L1 introduction and UCH-L3 depletion (Figure 3(B)). Furthermore, the CSC-like characteristics, such as the expression of pluripotency markers, sphere forming ability, and drug resistance, were also inhibited by NVP-BEZ235 in UCH-L1 overexpression and UCH-L3 knockdown cells (Figure 4). The cells were treated with a specific PI3 K inhibitor, LY294002, to further determine if the CSC-like properties increased by UCH-L1 overexpression or UCH-L3 depletion in RWPE1 cells occurred via the PI3 K/Akt signaling pathway. Similar to BEZ235, LY294002 decreased the number of spheres increased by UCH-L1 overexpression or UCH-L3 knockdown (Figure 5). These findings suggest that modulation of the CSC-like properties by UCH-L1 and UCH-L3 is mediated by the PI3 K/Akt signaling pathway.

### UCH-L1 and UCH-L3 differentially regulate the CSC-like behaviors through the PI3 K/Akt signaling pathway in DU145 cells

**Figure 6.**
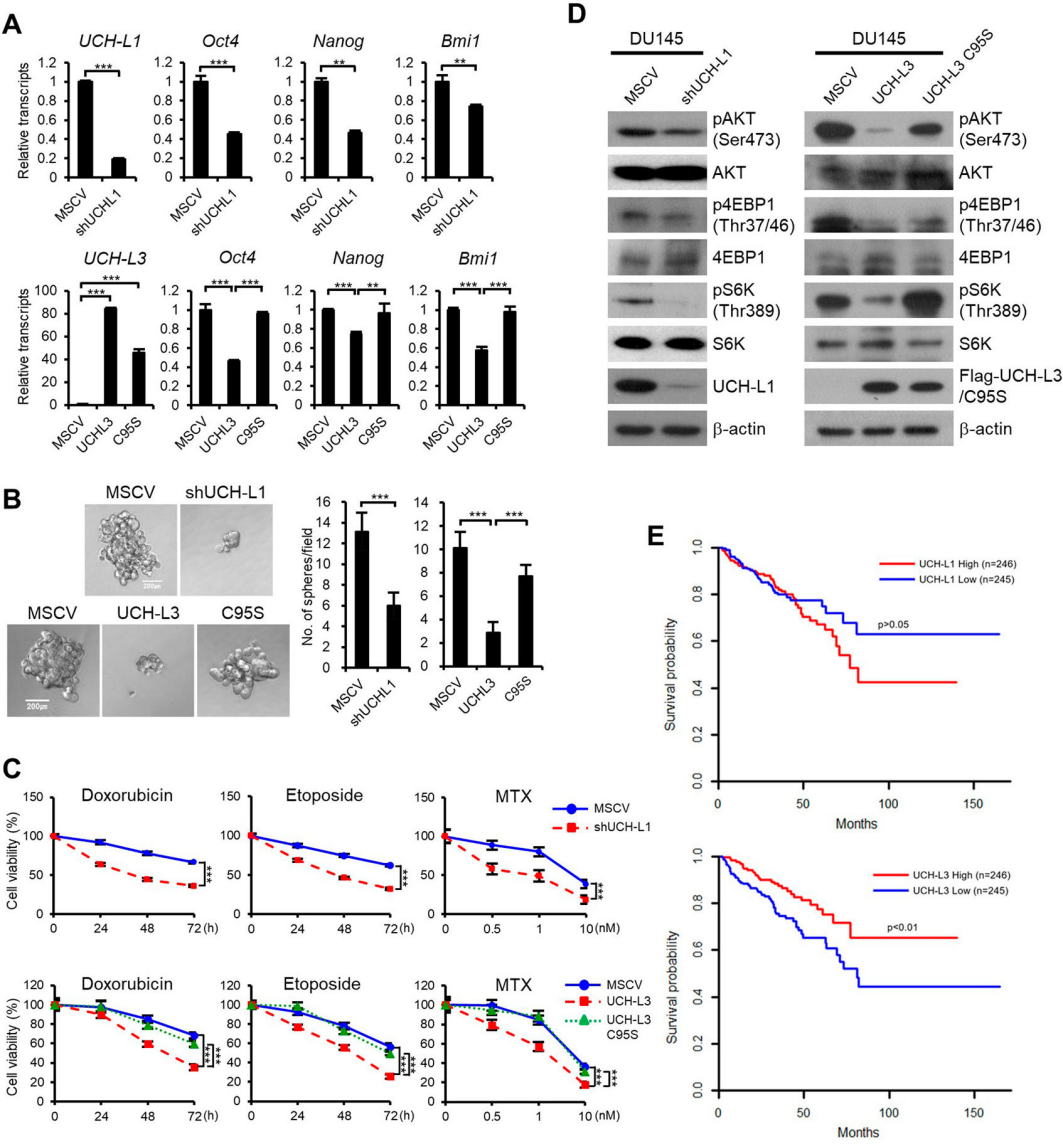
UCH-L1 and UCH-L3 regulate the CSC-like properties through the PI3K/Akt signaling pathway in DU145 cells. (A) Real-time quantitative RT-PCR analysis of *Oct4, Nanog*, and *Bmi1* in each DU145 stable cell. (B) Each DU145 stable cell was cultured in 96-well ultra-low attachment dishes, and the spheres formed after seven days were observed. The figure shows a representative image from each cell, and the scale bar corresponding to 200 μm was adjusted to all images. The number of spheres larger than 100 μm in diameter per field was counted. (C) The cell viability of each DU145 stable cell was monitored in a time-dependent manner after treating the cells with 0.5 μg/mL doxorubicin or 50 μM etoposide and in a dose-dependent manner after treating the cells with MTX for 48 h. (D) Western blotting analysis of several PI3 K/Akt signaling molecules, as indicated in each DU145 stable cell. (E) Kaplan-Meier disease-free survival curves for the high (n = 246) and low (n = 245) UCH-L1 or UCH-L3 expression groups. Each group was stratified by the median expression of UCH-L1 and UCH-L3. The values shown are the mean ± SD of three independent experiments, and the *p*-value was obtained from a Student’s t-test. *p < 0.05, **p < 0.01, ***p < 0.001.

In our previous study, UCH-L1 expression was high and UCH-L3 expression was low in highly metastatic prostate cells DU145, in contrast to RWPE1 cells (Jang et al. 2011; Song et al. 2014). To investigate the effects of UCH-L1 and UCH-L3 on the CSC-like characteristics in prostate cancer cells, a cell line with UCH-L1 knocked down, and a cell line that overexpressed UCH-L3 or UCH-L3 C95S were used in DU145 cells. In the UCH-L1 knockdown and UCH-L3 overexpressing cells, the expression of the CSC pluripotency markers, including Oct4, Nanog, and Bmi1, was decreased compared to the control (Figure 6(A)). UCH-L1 knockdown and UCH-L3 overexpression in DU145 cells decreased the sphere forming ability and resistance to chemicals, such as doxorubicin, etoposide, and MTX (Figure 6(B and C)). On the other hand, the CSC-like properties tested in this study were not decreased by UCH-L3 C95S overexpression.

Next we checked whether the regulation of CSC-like properties by UCH-L1 and UCH-L3 in DU145 is mediated through the PI3 K/Akt pathway. UCH-L1 knockdown and UCH-L3 overexpression inhibited the phosphorylation of the downstream targets of the PI3 K/Akt pathway, such as pAKT(Ser473), p4EBP1(Thr37/46), and pS6 K(Thr389). On the other hand, UCH-L3 C95S did not have any significant effects (Figure 6(D)). Overall, UCH-L1 activates the CSC-like properties in DU145 cells through the PI3 K/Akt pathway, whereas UCH-L3 has opposite effects.

As the relapse of CRPC is attributed to CSCs, we examined the relationship between the disease-free survival and the level of UCH-L1 and UCH-L3 in prostate cancer patients. The results of Kaplan-Meier analysis of 491 prostate cancer patients showed that patients expressing high UCH-L3 had higher disease-free survival compared to patients expressing relatively low UCH-L3 levels (*p* < 0.01, log-rank test) (Figure 6(E)). In the case of UCH-L1, although statistically insignificant, the disease-free survival rate tended to decrease in patients expressing high UCH-L1 levels (*p* > 0.05, log-rank test). The TCGA cohort studies suggested that the expression level of UCH-L1 and UCH-L3 affects the survival and recurrence of prostate cancers. Overall, these results showed that UCH-L1 and UCH-L3 could be effective therapeutic targets to eliminate the recurrence caused by CSCs in prostate cancers.

## Discussion

During tumor progression, EMT is important for cancer cells to invade and metastasize. Recently the functional link between EMT and the induction of CSC properties has been elucidated (Shibue and Weinberg 2017). CSCs remaining after a conventional cancer treatment caused drug resistance and the recurrence of many malignant cancers. Therefore, elucidating the regulators that function on the interplay of EMT and the CSC properties could provide a new therapeutic strategy for targeting CSCs (Tanabe et al. 2020).

UCH-L1 and UCH-L3 are DUBs classified as ubiquitin C-terminal hydrolases (UCHs). The implications of UCH-L1 and UCH-L3 in cancer development have been reported (Tezel et al. 2000; Miyoshi et al. 2006; Kim et al. 2009; Jang et al. 2011; Song et al. 2014). Several studies showed the promotion of cancer metastasis by UCH-L1 through EMT, including prostate and lung cancer cells (Jang et al. 2011; Liu et al. 2020). The association of CSC functions with UCH-L1 in glioma has also been reported (Sanchez-Diaz et al. 2017). The PI3 K/Akt signaling pathway in prostate cancer is important for maintaining CSCs (Dubrovska et al. 2009). In gastric cancer and lymphoma, the promotion of cancer metastasis caused by UCH-L1 via the PI3 K/Akt signaling pathway has been studied (Hussain et al. 2010; Gu et al. 2015). On the other hand, the association of the PI3 K/Akt pathway and the regulation of the CSC-like properties in prostate cancer cells has not been identified. Although the promotion of tumor progression by UCH-L3 has been identified in several reports (Miyoshi et al. 2006; Zhang et al. 2020), a previous study reported that UCH-L3 suppressed cancer metastasis through EMT inhibition in prostate cancer cells (Song et al. 2014). The view that UCH-L1 and UCH-L3 regulate EMT suggests that they might be involved in modulating the CSC-like properties of prostate cancer cells.

Our previous report identified that the UCH-L1 protein level was high in the metastatic prostate cancer cell line, DU145, and low in the benign tumor cell line, RWPE1 (Jang et al. 2011). In addition, the UCH-L3 protein expression pattern was the opposite of UCH-L1 (Song et al. 2014). The expression of UCH-L1 and UCH-L3 in RWPE1 or DU145 was reversed to determine the implications of these DUBs on the CSC-like properties. Either UCH-L1 overexpression or UCH-L3 depletion in RWPE1 cells augmented the CSC-like properties, including the induction of the pluripotent marker genes and the increase in sphere formation and drug resistance. In DU145 cells, the CSC-like properties were inhibited by UCH-L1 knockdown or UCH-L3 overexpression. Modulation of the CSC-like properties by UCH-L1 and UCH-L3 was mediated by the PI3 K/Akt pathway. Interestingly, Ouyang *et al*. reported that stabilization of the aryl hydrocarbon receptor by UCH-L3 maintains CSC-like properties in non-small cell lung cancers (Ouyang et al. 2020). This suggests that the function of UCH-L3 in regulating the CSC-like properties is dependent on the cell types and cellular context.

The detailed mechanism underlying the UCH-L1- and UCH-L3-mediated regulation of PI3 K/Akt signaling in the modulation of CSC properties remains to be elucidated. Epidermal growth factor receptor (EGFR) is a receptor tyrosine kinase that activates multiple downstream signaling cascades, including PI3 K/Akt. EGFR signaling plays a pivotal function in supporting the CSC properties, and aberrant EGFR expression is implicated in the progression of CRPC (Berger et al. 2006; Schneider and Yarden 2016). Recently, it was reported that UCH-L1 promotes cardiac hypertrophy through deubiquitination and stabilization of EGFR and activation of EGFR downstream mediators, including Akt (Bi et al. 2020). Thus, it may be possible that UCH-L1 mediated deubiquitination and stabilization of EGFR activates the PI3 K/Akt signaling in prostate cancer cells. Some kinases and phosphatases affecting Akt phosphorylation may also be involved in the PI3 K/Akt signaling regulation by UCH-L1 or UCH-L3. It has been reported that aberrant UCH-L1 expression increases Akt signaling by decreasing the antagonistic phosphatase PHLPP1 level, and this effect is independent of proteasome but dependent on the UCH-L1 catalytic activity (Hussain et al. 2010).

In this study, UCH-L1 and UCH-L3 differentially regulated the CSC-like properties of prostate cancer cells via the PI3 K/Akt signaling pathway. The CSC-like properties were enhanced by UCH-L1 but inhibited by UCH-L3. TCGA dataset analysis showed that a high level of UCH-L3 expression is positively correlated with the disease-free survival. By contrast, a high level of UCH-L1 expression had an inverse correlation with the survival rate. This suggests that these two DUBs could be promising diagnostic and therapeutic targets for treating prostate cancers. Although the differential effects of UCH-L1 and UCH-L3 on CSC-like behaviors have been identified, it is essential to identify the specific targets for these two deubiquitinating enzymes to achieve a deeper understanding underlying the molecular mechanism of prostate cancer progression.
